# Diagnostic Accuracy of Wearable ECG Devices for Atrial Fibrillation and ST-Segment Changes: A Systematic Review

**DOI:** 10.3390/diagnostics15243162

**Published:** 2025-12-11

**Authors:** Mara Sînziana Sîngeap, Luiza Elena Corneanu, Andrei Prodaniuc, Ivona Andreea Șova, Eric Oliviu Coșovanu, Ovidiu Rusalim Petriș

**Affiliations:** Grigore T. Popa University of Medicine and Pharmacy, 700115 Iași, Romania; mara-sinziana.singeap@umfiasi.ro (M.S.S.); prodaniuc.andrei@d.umfiasi.ro (A.P.); andreea-ivona_sova@umfiasi.ro (I.A.Ș.); cosovanu_eric-oliviu@d.umfiasi.ro (E.O.C.); ovidiu.petris@umfiasi.ro (O.R.P.)

**Keywords:** electrocardiography, wearable devices, smartwatch ECG, diagnostic accuracy, sensitivity, specificity

## Abstract

**Background**: Wearable electrocardiography (ECG) devices such as smartwatches offer a novel means for detecting cardiac arrhythmias, particularly atrial fibrillation (AF), and ST-segment abnormalities. Their role in complementing or replacing traditional ECG methods is being increasingly investigated. **Objective**: To evaluate the diagnostic performance (sensitivity, specificity) of wearable ECG devices in detecting AF and ST-segment changes, compared to 12-lead ECG as the gold standard. **Methods**: A systematic search was performed in PubMed, Scopus, and additionally, the SpringerLink platform was consulted up to June 2025, targeting open-access, English-language clinical studies from the last five years. Inclusion criteria: adult population, use of a wearable ECG device, 12-lead ECG comparator, and diagnostic accuracy reporting. Out of 145 records, 5 studies met the inclusion criteria. The systematic review protocol was not prospectively registered in PROSPERO due to the limited number of available studies and the exploratory nature of the topic, which focused on the most recent clinical evaluations of wearable ECG devices. However, the review strictly adhered to the PRISMA 2020 guidelines for systematic reviews to ensure methodological transparency and reproducibility. **Results**: Five studies encompassing a total of 1133 participants were incorporated into the analysis. Devices evaluated included Apple Watch (Series 4–6), KardiaMobile 6L, FibriCheck, Preventicus, and HUAMI dynamic ECG. Sensitivity ranged from 83% to 100%, and specificity from 79% to 100%. Algorithm improvements and repeated measurements significantly reduced inconclusive recordings. Multichannel ECG methods using smartwatches showed high agreement with 12-lead ECG in ST-elevation myocardial infarction detection. **Conclusions**: Wearable ECG devices demonstrate high diagnostic performance for AF and ST-segment abnormalities, especially in supervised environments. However, inconclusive recordings and algorithm limitations remain barriers to widespread clinical use. Real-world validation and algorithm refinement are needed for broader adoption.

## 1. Introduction

Atrial fibrillation (AF) is increasingly recognized as a global cardiovascular epidemic, with epidemiological studies indicating that between one in three and five individuals over the age of 45 will develop the condition during their lifetime. The sustained rise in AF prevalence, driven by population aging and improved survival from other cardiovascular diseases, positions it as a major contributor to the healthcare burden worldwide. In response, rhythm-control strategies, including catheter ablation, have gained prominence, with recent trials and meta-analyses (e.g., AFFIRM 2002 [1]) supporting their association with improved morbidity and mortality outcomes compared to rate-control alone. Consequently, contemporary guidelines recommend offering cardioversion to most patients with AF, particularly to evaluate the potential reversibility of tachycardia-induced cardiomyopathy.

This growing burden places substantial pressure on healthcare systems, not only in terms of the availability of ablation centers but also regarding accurate diagnosis and ongoing monitoring of this arrhythmia. While permanent AF can be easily identified through standard ECG, intermittent forms require dedicated detection strategies, ideally implemented before the first clinical manifestation of a thromboembolic complication such as stroke, an event for which AF increases the risk fivefold. Furthermore, effective cardioversion and decisions regarding long-term anticoagulation require precise knowledge of whether paroxysmal episodes of AF persist.

Another recent development involves the integration of smart technologies (smartwatches, wristbands, rings, etc.), part of the broader M-TECH (medical technology) concept, which are now capable of detecting AF through continuous monitoring. These tools allow patients to self-monitor their heart rhythm, not just through manual pulse checks, but via smart devices that use various algorithms for pulse wave analysis. The validity of such recordings must be confirmed by medical professionals through extended ECG monitoring techniques (such as ECG Holter or loop recorders), which are often limited by accessibility issues and the time-intensive nature of systematic interpretation. This has led to an expanded role for various healthcare professionals, particularly nurses, alongside cardiologists, internists, and general practitioners.

To explore the applicability of smart technology in the management of AF, we conducted a systematic review of published studies assessing the detection capabilities of smartwatches for cardiac arrhythmias, with a particular focus on AF. This analysis was extended to include the performance of these devices in detecting ST-segment abnormalities as well.

This systematic review aims to synthesize the available evidence on the diagnostic performance of wearable ECG devices in detecting AF and ST-segment abnormalities, compared to the reference standard of 12-lead ECG. The focus is on sensitivity, specificity, algorithm improvements, and the practical implications for clinical and unsupervised environments.

This study aimed to evaluate the diagnostic accuracy of wearable ECG devices using PICO-style research (Population: adults screened for AF or ST-segment abnormalities; Intervention: wearable ECG devices; Comparator: conventional 12-lead ECG or Holter monitoring; Outcome: sensitivity, specificity, and diagnostic accuracy).

However, despite the growing interest in wearable ECG technologies, their diagnostic performance, particularly in real-world clinical settings characterized by variable patient behaviors, comorbidities, and environmental factors, remains insufficiently investigated and poorly quantified, leaving a critical gap in the evidence base needed to support their widespread clinical adoption.

## 2. Materials and Methods

A comprehensive literature search was conducted using PubMed and Scopus databases up to June 2025. Additionally, the SpringerLink publisher platform was consulted to identify supplementary sources that may not have been retrieved through indexed databases. The search strategy utilized the following keywords: ‘Electrocardiography’ OR ‘EKG’ AND ‘Wearable Devices’ OR ‘Wearables’ OR ‘Smart Watches’ AND ‘Sensitivity and Specificity’. Only open-access, English-language clinical studies involving human adult participants (aged 18 years or older) were included. We restricted the search to open-access publications for several reasons. First, we adhered to our institutional access limitations. Second, articles without full-text availability cannot be thoroughly assessed, and, thus, we aimed to minimize the risk of incomplete or inaccurate data extraction. Third, limiting the search to open-access sources enhances transparency and reproducibility, enabling independent researchers to verify and reassess our findings. Additionally, we applied a language restriction by excluding studies not published in English. This decision was driven by several factors. Primarily, the inclusion of non-English articles without access to professional translation resources could have introduced interpretation errors and affected the consistency of data extraction. By restricting the review to English-language studies, we aimed to ensure the accuracy, transparency, and reproducibility of our findings, as all included texts were accessible and verifiable by other researchers. However, this restriction may have limited the scope of eligible publications and introduced potential selection bias, which is acknowledged and discussed in the limitations section. The inclusion criteria were: (1) use of a wearable ECG device; (2) comparison with a standard 12-lead ECG as the reference method; (3) assessment of diagnostic accuracy metrics such as sensitivity and specificity. Studies were excluded if they were preprints, reviews, pediatric studies, lacked a reference ECG comparator, or their primary objective focused on device usability, patient satisfaction, or algorithmic optimization rather than diagnostic accuracy.

**Figure 1:**
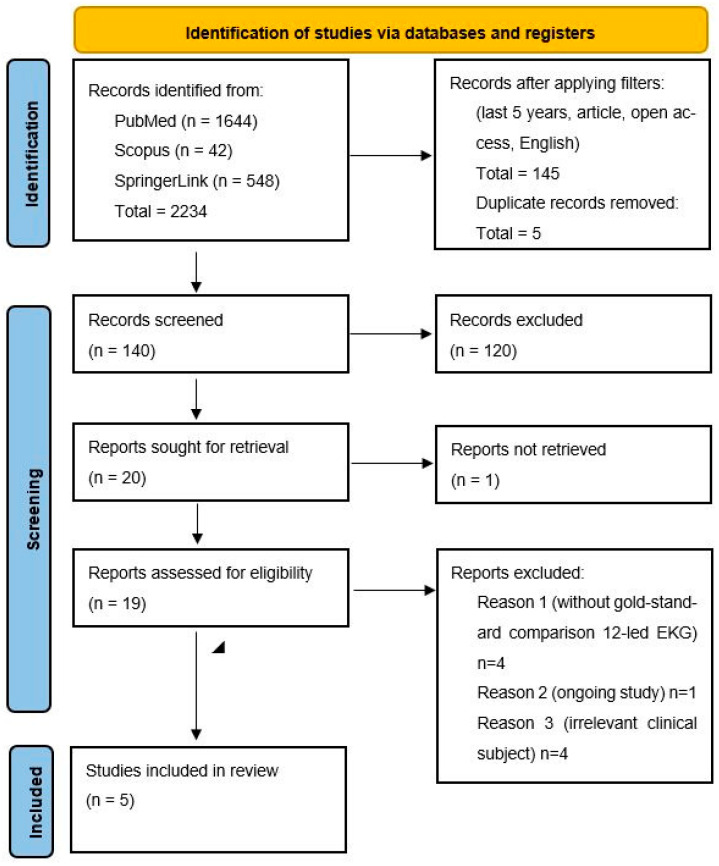
PRISMA flow diagram.

After filtering and duplicate removal, 140 articles remained for screening. Two independent reviewers evaluated titles and abstracts for relevance. Of these, 20 full texts were reviewed for eligibility, with 15 excluded for reasons including lack of ECG comparator, ongoing studies, and irrelevance. Ultimately, 5 studies were included in this systematic review. The PRISMA flow diagram (Figure 1) was used to illustrate the study selection process.

Additional information on the detailed search process and the methodology used for bias assessment can be found in Appendix A (Search Strategy) and Appendix B (Risk of Bias Evaluation).

The GRADE approach was not applied in this systematic review due to the small number of eligible studies (*n* = 5), as well as substantial clinical and methodological heterogeneity across device types, diagnostic algorithms, study populations, and outcome definitions. Additionally, since no quantitative meta-analysis was performed and the included studies did not evaluate a uniform intervention or comparator, the fundamental criteria required for a reliable GRADE assessment, such as consistency, precision, and directness of effect estimates, could not be adequately fulfilled. Therefore, applying GRADE in this context was considered neither appropriate nor methodologically justifiable, as it could have led to misleading certainty ratings.

This systematic review was conducted in accordance with the PRISMA 2020 guidelines (Supplementary Material). The review protocol was not registered in a public database.

## 3. Results

This systematic review included five studies (Table 1) that assessed the diagnostic performance of various wearable and consumer-grade ECG devices. The evaluated technologies comprised the Apple Watch (Series 4–6), KardiaMobile 6L (AliveCor, Mountain View, CA, USA), FibriCheck (Qompium NV, Hasselt, Belgium), Preventicus (Preventicus GmbH, Jena, Germany), and the HUAMI dynamic ECG wristband (Zepp Health, Hefei, China). All studies utilized the 12-lead ECG as the gold standard reference. Study populations varied in size (ranging from 74 to 723 participants) and included adult patients undergoing cardiac evaluation for AF, SR, or ST-segment abnormalities.

### 3.1. Diagnostic Accuracy of the Apple Watch

Multiple studies focused on the Apple Watch’s capacity to detect AF using its single-lead ECG algorithm. In the study by Pepplinkhuizen et al. (2022) [2], which included patients scheduled for electrical cardioversion, the Apple Watch Series 6 demonstrated a sensitivity of 94.6% and specificity of 100% when repeated recordings were permitted following inconclusive results. However, diagnostic performance declined considerably when unclassifiable or poor-quality tracings were treated as incorrect, resulting in a sensitivity of 66.2% and specificity of 73.4%. Notably, 27.9% of initial recordings were unclassifiable, a limitation mitigated by physician review, which reduced this rate to 1.6%. In all included studies, physician review of ECG tracings was applied as part of the reference standard (12-lead ECG or Holter monitoring) and not as a component of the index test.

### 3.2. Algorithmic Enhancements

In a large prospective study by Velraeds et al. (2023) [6], the performance of the Apple Watch’s standard algorithm was compared with a novel, enhanced algorithm in 723 participants. The standard version achieved 83% sensitivity and 79% specificity, with 19% of readings labeled as inconclusive. In contrast, the improved algorithm demonstrated significantly better performance, achieving 90% sensitivity, 92% specificity, and eliminating inconclusive results. This performance gain was particularly evident in patients with complex cardiac profiles, including conduction disturbances and frequent extrasystoles.

### 3.3. Comparative Evaluation of Multiple Devices

The study conducted by Wouters et al. (2025) [4] compared the diagnostic capabilities of the Apple Watch, KardiaMobile 6L, and two photoplethysmography (PPG)-based smartphone applications (FibriCheck and Preventicus) in 122 adult patients. All devices achieved 100% sensitivity for AF detection when the first good-quality recording was considered. Specificity was also high across devices, with the Apple Watch reaching 97.8% and FibriCheck achieving 98.9%. The usability and design of the Apple Watch were most preferred by patients; however, the rate of poor-quality or uninterpretable recordings remained notable at 10.7%.

### 3.4. Multichannel ECG Recording and ST-Segment Detection

Beyond AF detection, Spaccarotella et al. (2020) [5] explored the potential of the Apple Watch Series 4 to detect ST-segment changes indicative of acute coronary syndromes. By repositioning the device to mimic multichannel ECG acquisition, the smartwatch achieved a sensitivity of 93–94% and specificity of 92–100% for detecting both ST-elevation and non-ST-elevation myocardial infarction. Agreement with standard 12-lead ECG was high (Cohen’s kappa = 0.85–0.90), though these findings were dependent on precise manual device placement and physician interpretation, undermining the extrapolation of outcomes to settings without clinical oversight.

### 3.5. Diagnostic Performance of a Dynamic ECG Wristband

The HUAMI heart study [3] evaluated a wearable dynamic ECG wristband in 114 participants under varying physical conditions. The device exhibited excellent performance, with a sensitivity of 94.34% and a specificity of 100% in upright and post-exercise states. However, these findings should be interpreted with caution, given the small sample size and limited population diversity. Furthermore, the rate of inconclusive readings was minimal, indicating the potential for these devices to be used effectively in ambulatory or unsupervised contexts.

### 3.6. Cross-Study Observations and Limitations

Although diagnostic accuracy across studies was generally high, several limitations were consistently reported. Leading the list of challenges was the consistently high incidence of poor or inconclusive ECG tracings, which varied from 6% to nearly 28%, particularly among older individuals, those with pacemakers, or patients with reduced ECG signal amplitude [2,4]. In many studies, repeated measurements and interpretation by a qualified physician were necessary to obtain diagnostic-quality recordings, which may not be feasible in real-world settings [2,4,5].

Participants across the included studies were predominantly middle-aged to older adults, generally ranging from 50 to 75 years of age, with a balanced representation of men and women. The most frequently reported comorbidities were hypertension, diabetes mellitus, coronary artery disease, and structural heart disease, consistent with the typical atrial fibrillation risk profile. These characteristics indicate that the study populations largely reflect patients encountered in routine cardiovascular practice, supporting the external validity of the findings.

Additionally, most wearable algorithms were limited to distinguishing AF from SR. This limitation frequently resulted in false positives or unrecognized arrhythmias in the presence of atrial flutter, frequent premature contractions, or other supraventricular tachycardias [4,6]. Furthermore, many studies excluded patients with significant comorbidities or inadequate technological competence, raising concerns regarding the extent to which the results can be applied to broader populations [2,3,4,5].

### 3.7. Summary of Diagnostic Performance

The highest sensitivity was consistently reported in the Wouters et al. (2025) study [4], with all evaluated devices achieving 100% sensitivity for AF when a valid recording was obtained. The highest specificity was observed in the HUAMI study [3], which consistently demonstrated 100% specificity under all testing conditions. Enhanced algorithms and repeat measurements substantially improved diagnostic yield, though their need underscores the current challenges in achieving reliable, autonomous ECG monitoring using wearable devices [2,3,4].

## 4. Discussion

### 4.1. Principal Findings

The rapid evolution of wearable ECG technologies has transformed arrhythmia detection, particularly AF, by offering continuous, non-invasive, and user-friendly monitoring alternatives to conventional 12-lead ECG and Holter systems [7,8]. This systematic review synthesized recent evidence on the diagnostic performance of wearable ECG devices—especially the Apple Watch—highlighting their strong potential alongside persisting limitations that warrant further refinement [9,10]. Overall, the included studies consistently demonstrated high diagnostic accuracy under controlled conditions, with sensitivities up to 100% and specificities between 96% and 99% for AF detection, confirming reliable identification of sinus rhythm and low false-positive rates [4].

Moreover, algorithmic enhancements have proven instrumental in refining diagnostic accuracy. Velraeds et al. (2023) introduced a two-step algorithmic model that significantly outperformed the standard Apple Watch AF detection algorithm, raising sensitivity to 90%, specificity to 92%, and eliminating inconclusive recordings [6]. These improvements were particularly beneficial in patients with complex electrocardiographic backgrounds, such as those with baseline conduction abnormalities or frequent ectopy. Similarly, Gotlibovych et al. (2018) applied end-to-end deep learning algorithms on raw photoplethysmographic data from wearable devices, achieving near-perfect classification performance with an area under the curve (AUC) of 0.999 and minimal false detection rates, confirming the transformative role of artificial intelligence in wearable ECG interpretation [11].

### 4.2. Strengths and Limitations

Despite these advances, the studies also highlight several critical limitations. A major concern is the frequency of inconclusive or poor-quality recordings, especially when wearable devices are used in unsupervised or ambulatory settings. Pepplinkhuizen et al. (2022) observed that up to 27.9% of Apple Watch ECG recordings were unclassifiable on the first attempt, significantly reducing overall diagnostic utility [2]. Although repeated measurements and physician adjudication improved interpretability, they introduce logistical barriers and potential biases, particularly in patient self-monitoring contexts where clinical oversight is absent. Additionally, poor-quality recordings were more common in older adults [12] and individuals with pacemakers or movement disorders, revealing important gaps in device reliability among high-risk subgroups [13].

Another noteworthy limitation concerns the scope of rhythm classification. Most wearable ECG algorithms are designed primarily to distinguish AF from SR and lack the capacity to accurately identify other arrhythmias such as atrial flutter, supraventricular tachycardia, or premature atrial/ventricular contractions [14]. This restricted diagnostic scope may lead to misclassification, false positives, or missed opportunities for early intervention in patients with arrhythmias other than AF [15]. As a result, device outputs often require supplementary clinical review, reducing the scalability and autonomous utility of these technologies. Supervised device use in clinical settings typically yields greater diagnostic accuracy and fewer signal artifacts compared to unsupervised, home-based recordings, where patient adherence and motion interference can reduce data reliability.

Study design factors further constrain the generalizability of these findings to diverse clinical settings. Most investigations were conducted in controlled hospital-based or academic environments with selected patient cohorts—typically younger, technologically literate, and predominantly Caucasian individuals, thus limiting external validity [16,17,18,19].

These demographic characteristics limit the external validity of the results, as older adults, racially diverse groups, and individuals with multiple comorbidities remain underrepresented. Moreover, few studies explicitly evaluated long-term clinical outcomes, such as stroke incidence, hospital admissions, or changes in anticoagulation therapy decisions resulting from wearable device use [20,21,22]. Recent studies indicate that early atrial fibrillation detection through wearable ECG devices may facilitate prompt initiation of anticoagulation therapy and risk stratification, contributing to stroke prevention and improved long-term outcomes [21,22].

Moreover, the absence of a registered review protocol may be viewed as a limitation, as it restricts external verification of the predefined objectives, methods, and eligibility criteria. This may impact the reproducibility and transparency of the review process.

An important limitation of this systematic review is represented by the inclusion of only open-access literature, which may have led to the omission of potentially relevant non-open-access references. Another limitation of this review was the inclusion of only English-language studies. We acknowledge the potential for language bias and the limited generalizability of our findings. However, due to the lack of adequate translation resources, we accepted this limitation to ensure consistency and methodological quality throughout the review process.

Finally, the proprietary nature of commercial device algorithms and their frequent updates pose challenges to reproducibility and ongoing validation. Unlike open-source or regulated clinical tools, wearable technologies often lack transparency regarding the decision-making logic underlying rhythm classification. This absence of algorithmic clarity undermines independent evaluative processes, cross-device standardization, and regulatory oversight, all of which are critical for large-scale clinical implementation [23].

### 4.3. Clinical Implications

In sum, the body of evidence affirms that wearable ECG devices—especially those with integrated single-lead ECG functionality—offer high diagnostic performance for AF detection and represent a promising adjunct to traditional cardiac monitoring strategies. However, to fully leverage their clinical utility, further efforts are needed to address current limitations, including minimizing inconclusive recordings, expanding rhythm classification capabilities, enhancing usability in older or technologically vulnerable populations, and validating their effectiveness in real-world settings. Collaborative initiatives between clinicians, technologists, and regulatory bodies will be essential to bridge these gaps and advance wearable ECG technologies from experimental tools to standard components of cardiovascular care.

Nurses also play a vital role in ECG monitoring and preliminary data evaluation, ensuring proper electrode placement, signal quality, and prompt identification of critical abnormalities. Through their technical expertise and clinical vigilance, they contribute to accurate ECG interpretation and early medical intervention. Integrating smart technologies with nurse-led care can therefore enhance the early detection and precise diagnosis of atrial fibrillation, a concept well supported by existing literature.

## 5. Conclusions

Wearable ECG devices show encouraging potential as adjunct tools for the detection of atrial fibrillation and, to a more limited extent, ST-segment abnormalities. However, the current evidence remains restricted by the small number of available studies, variability in device performance, and inconsistent signal quality. Diagnostic accuracy outside controlled environments is influenced by user-dependent factors, algorithmic limitations, and the high proportion of inconclusive recordings, particularly in older or less digitally literate populations [24,25].

Evidence supporting the use of wearable devices for arrhythmias other than atrial fibrillation is currently insufficient, as most studies focus exclusively on AF detection and provide limited validation for alternative rhythm disorders [24,25].

Therefore, while wearable ECG technologies represent a promising complement to traditional diagnostic modalities, their clinical integration requires further standardization, broader real-world validation, and careful professional oversight. Larger, methodologically robust studies are needed to clarify their diagnostic scope across diverse populations and arrhythmic conditions [24,25].

## 6. Future Research Directions

The expanding landscape of wearable electrocardiographic technologies presents a wealth of opportunities for innovation, clinical application, and public health impact. Building upon the strong diagnostic performance observed in current studies [26], future research and development must address several strategic directions to fully unlock the potential of wearable devices in cardiovascular care. Key areas include enhancing device accuracy and usability [27], improving data integration with clinical systems, and addressing regulatory and privacy concerns to ensure patient safety and trust [10].

Furthermore, large-scale clinical trials and longitudinal studies are essential to validate real-world effectiveness and support evidence-based implementation.

First, there is an urgent need to broaden the demographic scope of validation studies. Most existing research has been conducted in homogeneous, often younger, and predominantly Caucasian populations within supervised clinical settings [28]. Future investigations should prioritize inclusivity by enrolling diverse patient cohorts, including older adults, individuals from different ethnic and socioeconomic backgrounds, and those with complex comorbidities. These studies should also be conducted in real-world, unsupervised environments to assess usability, adherence, and diagnostic reliability under routine conditions [29].

Second, the diagnostic capabilities of wearable algorithms must be expanded beyond binary classification of AF versus SR. Current algorithms lack sensitivity to other clinically significant arrhythmias, such as atrial flutter, supraventricular tachycardia, or frequent ectopic beats [30]. Future iterations should incorporate multi-class rhythm discrimination and real-time anomaly detection powered by advanced machine learning frameworks [31]. Moreover, integration of multi-modal sensor data, such as combining ECG with PPG, accelerometry, and even continuous oxygen saturation, may improve diagnostic accuracy and contextual understanding of cardiac events [32].

Third, reducing the rate of inconclusive or poor-quality recordings remains a critical goal. Strategies to mitigate this issue include improving signal acquisition hardware, refining artifact reduction algorithms, and implementing user guidance systems with real-time feedback on recording quality [33]. Adaptive learning algorithms that tailor instruction and detection parameters based on individual user characteristics (e.g., age, motion, skin tone, tremor frequency) could significantly enhance usability and data quality [34].

Fourth, future research should explore the longitudinal clinical impact of wearable-driven detection. While current studies demonstrate high diagnostic performance, few have assessed whether early arrhythmia detection translates into better clinical outcomes such as reduced stroke incidence, fewer hospitalizations, or improved survival [16]. Randomized controlled trials examining intervention strategies triggered by wearable findings (e.g., anticoagulation initiation or rhythm management) are essential to establish evidence-based clinical pathways, as has been performed for other digital health technologies like electronic blood pressure monitors regulated by the FDA [35].

Fifth, there is a compelling need for transparent algorithm design and regulatory standardization. Proprietary diagnostic models limit reproducibility and external validation [36]. Collaborative frameworks involving manufacturers, regulatory agencies, and clinical stakeholders should aim to develop open, validated diagnostic models and standardized performance metrics that can facilitate cross-device comparison and certification [37].

Finally, as wearable ECGs become increasingly integrated into digital health ecosystems, interoperability—following the M-TECH (Modular, Transparent, Efficient, Connected, and Human-centric) concept—with electronic health records and telemedicine platforms must be prioritized. Integrated data exchange will enable clinicians to receive alerts, review arrhythmia episodes, and act on time-sensitive information [38]. This integration also supports the development of centralized population health surveillance systems, capable of identifying epidemiological trends and informing public health interventions.

Wearable ECG technologies are gaining prominence as effective tools for the early detection and continuous monitoring of AF and ST-segment abnormalities. With high diagnostic accuracy and growing accessibility, these devices offer strong potential to complement traditional cardiac diagnostics, particularly in ambulatory and patient-led settings. Nonetheless, their broader clinical adoption depends on addressing several limitations. These include the need for validation in diverse populations, expansion of diagnostic algorithms beyond AF and SR, reduction of inconclusive recordings, and demonstration of real-world clinical impact. Equally important are transparent algorithm development, regulatory standardization, and seamless integration with digital health systems. By advancing along these dimensions, wearable ECG technologies may become integral components of a modern, preventive, and patient-centered cardiovascular care paradigm.

In summary, the future of wearable ECG technology hinges on inclusive validation, algorithmic expansion, artifact minimization, clinical impact assessment, transparent development, and health system integration. By addressing these directions, wearable devices will be well-positioned to play a transformative role in cardiovascular diagnostics and preventive care across diverse healthcare settings.

## Figures and Tables

**Table 1:** Comparative table of portable ECG devices.

Study	Study Design	Device	Population	Reference Standard	TP (True Positives)	FP (False Positives)	TN (True Negatives)	FN (False Negatives)	Clinical Setting	Sensitivity	Specificity	Accuracy	Comments
Pepplinkhuizen et al. (2022) [2]	Prospective diagnostic validation study	Apple Watch (Single-lead ECG)	74 patients with AF (mean age 67.1)	12-lead ECG	43	0	47	3	Inpatient cardiology ward; simultaneous smartwatch + 12-lead ECG recordings in hospitalized patients	93–94.6%	96.5–100%	Not specified but inferred from sensitivity/specificity	High accuracy for AF detection, limited by unclassifiable readings.
Fu and Li (2021) [3]	Cross-sectional observational study	Wearable dynamic ECG recorder (Huami)	114 outpatients (53 with AF)	12-lead ECG	47	0	61	6	Community/outpatient screening; AF vs. SR recordings under rest and mild activity	88.68–94.34%	100%	94.74–97.37%	Excellent sensitivity/specificity across body positions; feasible for remote use.
Wouters et al. (2025) [4]	Comparative validation study	Apple Watch, KardiaMobile 6L, FibriCheck, Preventicus	122 participants (30 with AF)	12-lead ECG	25	2	95	0	Outpatient cardiology clinic; resting ECG comparison in patients undergoing routine rhythm evaluation	100% for all devices	96.4–98.9%	High (97.4–99.2%)	All devices performed well; the Apple Watch was favored for usability.
Spaccarotella et al. (2020) [5]	Feasibility and diagnostic concordance study	Apple Watch (Multichannel ECG)	100 participants (MI patients and controls)	12-lead ECG	50	3	43	4	Emergency department/cath-lab setting; patients presenting with chest pain and suspected myocardial ischemia	84–94%	92–100%	High (Cohen’s k: 0.85–0.90)	Accurate for ST-segment changes with high agreement; requires physician guidance.
Velraeds et al. (2023) [6]	Algorithm development and validation study	Apple Watch (Enhanced AF detection algorithm)	723 patients (21% with AF)	12-lead ECG	26	9	106	3	Hospital inpatient cohort (cardiology department); mixed ECG abnormalities including AF, PACs, PVCs	90%	92%	Improved (91.67%) vs. original Apple algorithm (79.86%)	Improved diagnostic performance with an enhanced algorithm; eliminated inconclusive results.

## Data Availability

No new data were created or analyzed in this study. Data sharing is not applicable to this article.

## Supplementary Materials

The following are available online at https://www.mdpi.com/2075-4418/15/24/3162/s1, PRISMA 2020 Checklist [39].

## Abbreviations

The following abbreviations are used in this manuscript:

ECG	Electrocardiography
AF	Atrial fibrillation
SR	Sinus rhythm
M-TECH	Medical technology
PPG	Photoplethysmography
AUC	Area under the curve
TP	True positives
FP	False positives
TN	True negatives
FN	False negatives

## Appendix A

The literature search was conducted during May–June 2025 using two electronic databases: PubMed and Scopus. In addition, the SpringerLink publisher platform was consulted in order to ensure the retrieval of potentially relevant sources not found in indexing databases. The search strategy utilized the following keywords: ‘Electrocardiography’ OR ‘EKG’ AND ‘Wearable Devices’ OR ‘Wearables’ OR ‘Smart Watches’ AND ‘Sensitivity and Specificity’. An initial search without any filters yielded a total of 2234 articles. After applying filters to include only studies published within the last five years, open-access, and written in English, 688 articles remained. A manual screening was then performed to include only specific study types: Adaptive Clinical Trial, Classical Article, Clinical Study, Clinical Trial, Comparative Study, Controlled Clinical Trial, Corrected and Republished Article, Evaluation Study, Multicenter Study, Observational Study, Randomized Controlled Trial, Twin Study, and Validation Study. This refinement resulted in 145 articles.

Following duplicate removal, 140 unique articles were retained for title and abstract screening. Out of the total records identified and screened, 20 articles were sought for full-text retrieval. One article was excluded at this stage due to inaccessibility, resulting in 19 full-text articles that were assessed for eligibility. During this assessment, several articles were excluded based on predefined criteria: four studies were excluded due to the absence of a gold-standard comparator (12-lead ECG), one study was excluded because it was still ongoing, and four studies were excluded due to an irrelevant clinical focus. Following these exclusions, a total of five studies met all inclusion criteria and were included in the final systematic review.

## Appendix B

A Risk of Bias assessment was conducted across the studies included in our analysis to evaluate the methodological quality and potential limitations. The evaluation focused on patient selection, the index test, and the reference standard. Results showed variability in the level of bias across the included works (Table A1). While some exhibited a consistently low risk across all evaluated domains, others demonstrated moderate levels of bias. Two reviewers independently evaluated each study based on critical design domains. Any discrepancies in their assessments were addressed through discussion, and if consensus could not be reached, a third reviewer was consulted.

**Table A1:** Risk of Bias Assessment for Included Studies.

Author	Study Design	Risk of Bias Tool Used	Overall Risk of Bias	Justification
Pepplinkhuizen et al. (2022) [2]	Prospective diagnostic validation study	ROBINS I	Moderate	This study confirms the high accuracy of the Apple Watch ECG for detecting AF in a clinical setting. While unclassified results slightly complicate interpretation, the study is generally well-conducted with only a low to moderate risk of bias. The findings are relevant for validating the device’s utility in AF detection among patients undergoing cardioversion.
Fu and Li (2021) [3]	Cross-sectional observational study	ROBINS I	Low	The study shows promising diagnostic performance of a wearable ECG device for AF screening, but suffers from a low-to-moderate risk of bias due to non-random participant selection.
Wouters et al. (2025) [4]	Comparative validation study	ROBINS I	Low	This validation study offers a robust comparison across several consumer wearable devices for AF detection, showing high diagnostic accuracy overall. However, the low risk of bias is primarily due to participant selection.
Spaccarotella et al. (2020) [5]	Feasibility and diagnostic concordance study	ROBINS I	Moderate	The study effectively demonstrates the technical feasibility of recording multichannel ECG with a smartwatch and detecting ST-segment changes. However, it carries a moderate risk of bias due to the selective inclusion of confirmed myocardial infarction patients and the absence of confounder control.
Velraeds et al. (2023) [6]	Algorithm development and validation study	ROBINS I	Moderate	This study proposes a meaningful algorithmic improvement in AF detection using Apple Watch ECG data. While the design included both development and validation cohorts, a moderate risk of bias remains due to possible selection bias and unadjusted confounding factors. Nonetheless, the study provides useful evidence to enhance smartwatch-based AF diagnosis.
