# Supplementary material for: Diagnostic Accuracy of Wearable ECG Devices for Atrial Fibrillation and ST-Segment Changes: A Systematic Review
Source: Diagnostics (Basel). 2025 Dec 11;15(24):3162. doi: 10.3390/diagnostics15243162 (PMC12731301; doi:10.3390/diagnostics15243162)
Supplement: Supplementary file 1 [file diagnostics-15-03162-s001.zip › diagnostics-3974933-supplementary.pdf]

PRISMA 2020 Checklist

Section and Topic	Item #	Checklist item	Location where item is reported
**TITLE**			
Title	1	Identify the report as a systematic review.	Pg. 1<br>Diagnostic Accuracy of Wearable ECG Devices for Atrial Fibrillation and ST-Segment Changes: A Systematic Review
**ABSTRACT**			
Abstract	2	See the PRISMA 2020 for Abstracts checklist.	Pg. 1<br>The abstract was structured in accordance with the PRISMA 2020 for Abstracts checklist to ensure transparency, methodological clarity, and alignment with current standards for reporting systematic reviews.
**INTRODUCTION**			
Rationale	3	Describe the rationale for the review in the context of existing knowledge.	Pg. 2<br>The authors describe the increasing prevalence and clinical burden of atrial fibrillation, the limitations of conventional ECG monitoring in detecting intermittent arrhythmias, and the emerging role of wearable ECG devices. They also highlight gaps in the literature regarding the diagnostic accuracy of such devices, justifying the need for a systematic synthesis of current evidence.
Objectives	4	Provide an explicit statement of the objective(s) or question(s) the review addresses.	Pg. 2<br>The objective is clearly articulated: to assess the diagnostic accuracy, specifically sensitivity and specificity, of wearable ECG devices in identifying atrial fibrillation and ST-segment abnormalities, using 12-lead ECG as the reference standard.
**METHODS**			
Eligibility criteria	5	Specify the inclusion and exclusion criteria for the review and how studies were grouped for the syntheses.	Pg. 2-4<br>The eligibility criteria are clearly outlined in the *Materials and Methods* section. Studies were included if they involved adult participants (≥18 years), used wearable ECG devices, compared findings with a 12-lead ECG as reference, and reported diagnostic accuracy metrics (sensitivity and specificity). Exclusion criteria comprised preprints, reviews, pediatric populations, absence of a standard comparator, and lack of relevance to the review objectives. For synthesis purposes, studies were grouped according to the type of device assessed (e.g., Apple Watch, KardiaMobile, HUAMI) and the clinical target (atrial fibrillation or ST-segment abnormalities), as detailed in Table 1 and the *Results* section.
Information sources	6	Specify all databases, registers, websites, organisations, reference lists and other sources searched or consulted to identify studies. Specify the date when each source was last searched or consulted.	Pg. 2, Appendix 1<br>Three electronic databases—PubMed, Scopus, and SpringerLink—were systematically searched to identify relevant studies. The last search was conducted in June 2025. Additional details regarding the search process are provided in *Appendix 1*.
Search strategy	7	Present the full search strategies for all databases, registers and websites, including any filters and limits used.	Pg. 3, Appendix 1<br>The full electronic search strategies, including search terms and applied filters (language, publication type, time frame, and access), are provided in *Appendix 1*. Searches were conducted in PubMed, Scopus, and SpringerLink using combinations of terms related to electrocardiography, wearable devices, and diagnostic accuracy. Filters were applied to include only English-language, open-access clinical studies published within the past five years.
Selection process	8	Specify the methods used to decide whether a study met the	Pg. 3, Appendix 1

Section and Topic	Item #	Checklist item	Location where item is reported
		inclusion criteria of the review, including how many reviewers screened each record and each report retrieved, whether they worked independently, and if applicable, details of automation tools used in the process.	The selection process was conducted by two independent reviewers who screened all titles and abstracts for relevance. Full texts of potentially eligible studies were assessed independently based on predefined inclusion and exclusion criteria. Discrepancies were resolved through discussion, and when necessary, a third reviewer was consulted. No automation tools were used during the selection process.
Data collection process	9	Specify the methods used to collect data from reports, including how many reviewers collected data from each report, whether they worked independently, any processes for obtaining or confirming data from study investigators, and if applicable, details of automation tools used in the process.	Pg. 11<br>Data were extracted independently by two reviewers (L.E.C. and E.O.C.) using a standardized data collection process, as stated in the Author Contributions section. No automation tools were used, and no attempts were made to contact study authors for additional data.
Data items	10a	List and define all outcomes for which data were sought. Specify whether all results that were compatible with each outcome domain in each study were sought (e.g. for all measures, time points, analyses), and if not, the methods used to decide which results to collect.	Pg. 3-5<br>The primary outcomes of interest were diagnostic accuracy metrics, including sensitivity, specificity, and overall accuracy. Secondary outcomes included the rate of inconclusive recordings and algorithmic performance improvements. These were extracted as reported in each study. The review focused on the most relevant and complete data sets provided per device and arrhythmia type. No restrictions were applied to measurement time points or analytic methods unless reporting was insufficient.
	10b	List and define all other variables for which data were sought (e.g. participant and intervention characteristics, funding sources). Describe any assumptions made about any missing or unclear information.	Pg. 3-4, Table 1<br>In addition to diagnostic outcomes, the following study-level variables were extracted: study design, type of wearable ECG device, population size and characteristics (e.g., presence of AF or MI), type of ECG algorithm (standard or enhanced), and ECG modality (single-lead or multichannel). No data were extracted regarding funding sources of the included studies. When outcome details were unclear or missing, only the information explicitly reported by the authors was retained; no assumptions or imputations were made.
Study risk of bias assessment	11	Specify the methods used to assess risk of bias in the included studies, including details of the tool(s) used, how many reviewers assessed each study and whether they worked independently, and if applicable, details of automation tools used in the process.	Pg. 12, Appendix 2<br>Risk of bias was assessed for all included studies using the ROBINS-I tool, which is appropriate for non-randomized diagnostic evaluations. Two reviewers independently conducted the assessments. Disagreements were resolved by discussion, and a third reviewer was consulted if consensus was not reached. No automation tools were used in the assessment process.
Effect measures	12	Specify for each outcome the effect measure(s) (e.g. risk ratio, mean difference) used in the synthesis or presentation of results.	Pg. 3-5<br>For each included study, the effect measures extracted and synthesized were sensitivity, specificity, and overall diagnostic accuracy, expressed as percentages. These metrics were reported directly as provided by the original articles. No pooled effect estimates or statistical comparisons (e.g., risk ratios, mean differences) were conducted due to heterogeneity in study design and outcome reporting.
Synthesis methods	13a	Describe the processes used to decide which studies were eligible for each synthesis (e.g. tabulating the study intervention characteristics and comparing against the planned groups for each synthesis (item #5)).	Pg. 3-5<br>Eligibility for synthesis was determined based on whether each study met the predefined inclusion criteria (as outlined in Item 5), and whether it reported sensitivity and specificity for atrial fibrillation and/or ST-segment abnormalities using a wearable ECG device. Studies were tabulated and grouped by device type and diagnostic target (AF or ST changes) in Table 1, which guided the narrative synthesis and comparative evaluation.

Section and Topic	Item #	Checklist item	Location where item is reported
	13b	Describe any methods required to prepare the data for presentation or synthesis, such as handling of missing summary statistics, or data conversions.	No data conversions or transformations were applied. Sensitivity, specificity, and accuracy values were extracted as reported by the original studies. No missing summary statistics were imputed, and all results were presented descriptively without statistical synthesis or standardization.
	13c	Describe any methods used to tabulate or visually display results of individual studies and syntheses.	Pg. 4-5<br>Results from individual studies were summarized in a structured comparative table (Table 1), which included study design, device evaluated, sample characteristics, reference standard, diagnostic performance metrics, and author commentary. Additionally, the PRISMA flow diagram (Figure 1) was used to visually represent the study selection process. No statistical plots (e.g., forest plots) were used, as no meta-analysis was conducted.
	13d	Describe any methods used to synthesize results and provide a rationale for the choice(s). If meta-analysis was performed, describe the model(s), method(s) to identify the presence and extent of statistical heterogeneity, and software package(s) used.	Due to the small number of eligible studies (n=5) and the substantial heterogeneity in device types, algorithms, populations, and outcome reporting, no meta-analysis was performed. Instead, a structured narrative synthesis was conducted, grouping results by device and diagnostic focus (atrial fibrillation vs. ST-segment abnormalities). This approach was chosen to preserve the clinical context and to allow for descriptive comparison of diagnostic accuracy metrics. No statistical models, heterogeneity assessments, or synthesis software were used.
	13e	Describe any methods used to explore possible causes of heterogeneity among study results (e.g. subgroup analysis, meta-regression).	No formal statistical methods were used to explore heterogeneity, such as subgroup analysis or meta-regression. However, sources of clinical and methodological heterogeneity were explored narratively by comparing diagnostic performance across device types, algorithm versions, ECG modalities (e.g., single-lead vs. multichannel), and patient populations. These comparisons are described in the *Results* and *Discussion* sections to contextualize differences in sensitivity, specificity, and usability.
	13f	Describe any sensitivity analyses conducted to assess robustness of the synthesized results.	No sensitivity analyses were conducted due to the limited number of included studies and the narrative nature of the synthesis. All studies that met the predefined inclusion criteria were retained in the final review, regardless of methodological variability.
Reporting bias assessment	14	Describe any methods used to assess risk of bias due to missing results in a synthesis (arising from reporting biases).	No formal assessment of reporting bias (e.g., publication bias) was conducted due to the small number of included studies and the narrative nature of the synthesis. The review was limited to open-access, English-language publications indexed in major databases, which may introduce some selection bias; however, the risk of missing key results was considered minimal in this context.
Certainty assessment	15	Describe any methods used to assess certainty (or confidence) in the body of evidence for an outcome.	No formal methods (e.g., GRADE) were used to assess the certainty or confidence in the body of evidence for the reported outcomes. Given the small number of included studies and the narrative nature of the synthesis, the authors considered a structured certainty assessment to be beyond the scope of this review.
**RESULTS**			
Study selection	16a	Describe the results of the search and selection process, from the number of records identified in the search to the number of studies included in the review, ideally using a flow diagram.	The results of the search and selection process are clearly described in the *Materials and Methods* and *Appendix 1*, and visually summarized in the PRISMA 2020 flow diagram (Figure 1). From an initial 2,234 records, 688 remained after filters were applied, and 140 were retained after duplicate removal. After title and abstract screening, 20 full-text articles were reviewed, and 5 met all eligibility criteria and were included in the final synthesis.
	16b	Cite studies that might appear to meet the inclusion criteria, but	The review explains that several studies were excluded after full-text assessment due to

Section and Topic	Item #	Checklist item	Location where item is reported
		which were excluded, and explain why they were excluded.	reasons such as absence of a 12-lead ECG comparator, ongoing study status, or irrelevant focus. However, these studies are not individually cited in the manuscript.
Study characteristics	17	Cite each included study and present its characteristics.	All five included studies are clearly cited in the *Results* section and fully referenced in the bibliography. Their key characteristics—including study design, device evaluated, population, reference standard, and diagnostic performance—are summarized in Table 1. Additional narrative description is provided for each study in the *Results* section.
Risk of bias in studies	18	Present assessments of risk of bias for each included study.	Risk of bias assessments for all included studies were conducted using the ROBINS-I tool and are presented in *Appendix 2*. Each study was evaluated across key design domains, and the overall risk of bias was classified as low or moderate, with accompanying justifications. Two reviewers independently performed the assessments, and discrepancies were resolved by discussion or third-party consultation.
Results of individual studies	19	For all outcomes, present, for each study: (a) summary statistics for each group (where appropriate) and (b) an effect estimate and its precision (e.g. confidence/credible interval), ideally using structured tables or plots.	Summary measures for diagnostic accuracy—namely sensitivity, specificity, and overall accuracy—are presented for each included study in Table 1 and described narratively in the *Results* section. However, confidence intervals and group-level summary statistics (e.g., number of true positives, false negatives) were not consistently reported across the original studies and therefore are not presented. The effect estimates are shown as point values, as provided by the authors.
Results of syntheses	20a	For each synthesis, briefly summarise the characteristics and risk of bias among contributing studies.	The narrative synthesis was structured by device type and arrhythmia target (e.g., Apple Watch for AF, multichannel ECG for ST changes). Across the five included studies, sample sizes ranged from 74 to 723 participants, with populations typically composed of adults undergoing cardiac evaluation. While diagnostic accuracy was consistently high, methodological variability was observed in terms of study design, algorithm type, and ECG modality. Risk of bias was generally low to moderate, as assessed using ROBINS-I and detailed in Appendix 2.
	20b	Present results of all statistical syntheses conducted. If meta-analysis was done, present for each the summary estimate and its precision (e.g. confidence/credible interval) and measures of statistical heterogeneity. If comparing groups, describe the direction of the effect.	No statistical syntheses or meta-analyses were conducted due to the limited number of studies and substantial heterogeneity in device types, study designs, and reported outcomes. As such, no pooled effect estimates, confidence intervals, or measures of statistical heterogeneity (e.g., I^2) are reported. Results were synthesized narratively by device and arrhythmia type.
	20c	Present results of all investigations of possible causes of heterogeneity among study results.	No formal statistical analyses were conducted to investigate heterogeneity. However, potential sources of clinical and methodological heterogeneity were explored narratively. Differences in device type, algorithm version, ECG lead configuration, and patient populations were qualitatively assessed to explain variations in diagnostic performance across studies. These factors are discussed in the *Results* and *Discussion* sections.
	20d	Present results of all sensitivity analyses conducted to assess the robustness of the synthesized results.	No sensitivity analyses were conducted due to the small number of included studies and the narrative nature of the synthesis. All eligible studies were retained in the review, and no subgroup exclusions or re-analyses were performed.
Reporting biases	21	Present assessments of risk of bias due to missing results (arising from reporting biases) for each synthesis assessed.	No formal assessments of risk of bias due to missing results (e.g., publication bias) were performed, as no statistical synthesis or meta-analysis was conducted. Nonetheless, the restriction to open-access, English-language articles may have introduced a degree of reporting bias. This limitation is acknowledged in the discussion of study selection.
Certainty of evidence	22	Present assessments of certainty (or confidence) in the body of evidence for each outcome assessed.	Certainty of evidence was not formally assessed using tools such as GRADE. Given the narrative nature of the synthesis, the limited number of included studies, and

Section and Topic	Item #	Checklist item	Location where item is reported
			heterogeneity across device types and outcome measures, a structured certainty appraisal was deemed beyond the scope of this review.
**DISCUSSION**			
Discussion	23a	Provide a general interpretation of the results in the context of other evidence.	The *Discussion* section provides a comprehensive interpretation of the review findings in relation to existing evidence. The results support the diagnostic utility of portable ECG devices—particularly Apple Watch and KardiaMobile—in detecting atrial fibrillation with high sensitivity and specificity. These findings are consistent with previous clinical studies and meta-analyses. The discussion further integrates epidemiological considerations and emphasizes the evolving role of wearable technologies and multidisciplinary healthcare teams in rhythm disorder management.
	23b	Discuss any limitations of the evidence included in the review.	The body of evidence included in the review is limited by the small number of available studies, heterogeneous methodologies, and inconsistent reporting of diagnostic metrics (e.g., confidence intervals). Device types, ECG lead configurations, and algorithm versions varied across studies, complicating direct comparisons. Additionally, the included populations were often pre-selected (e.g., post-cardioversion), which may limit generalizability. Risk of bias assessments indicated low to moderate quality in most studies, as detailed in Appendix 2.
	23c	Discuss any limitations of the review processes used.	Several limitations of the review process should be acknowledged. First, the search strategy was limited to open-access, English-language publications, potentially introducing language and publication bias. Second, while data screening and extraction were conducted independently by two reviewers, only a limited number of discrepancies required third-party resolution, which may underrepresent disagreements. Third, no automation tools or machine-assisted selection methods were used, and all synthesis steps were performed manually. Finally, due to heterogeneity, no meta-analyses were conducted, which may limit the statistical integration of findings.
	23d	Discuss implications of the results for practice, policy, and future research.	The findings support the integration of portable ECG devices into clinical practice as feasible tools for early detection of atrial fibrillation and potentially ischemic changes. Their use may empower patients in self-monitoring and reduce delays in diagnosis, particularly when validated by trained medical personnel, such as specialized nurses. From a health policy perspective, structured pathways for triaging wearable ECG data and ensuring interoperability with electronic health systems are warranted. Future research should focus on larger, more homogeneous study designs, expansion to other arrhythmias, and the development of AI-enhanced diagnostic algorithms to further improve accuracy and usability.
**OTHER INFORMATION**			
Registration and protocol	24a	Provide registration information for the review, including register name and registration number, or state that the review was not registered.	This review was not registered.
	24b	Indicate where the review protocol can be accessed, or state that a protocol was not prepared.	This review has not a prepared protocol.
	24c	Describe and explain any amendments to information provided at registration or in the protocol.	-
Support	25	Describe sources of financial or non-financial support for the	This review did not receive any financial or non-financial support. No funders or sponsors

PRISMA 2020 Checklist

Section and Topic	Item #	Checklist item	Location where item is reported
		review, and the role of the funders or sponsors in the review.	were involved in the design, conduct, or reporting of the review.
Competing interests	26	Declare any competing interests of review authors.	The authors declare no competing interests.
Availability of data, code and other materials	27	Report which of the following are publicly available and where they can be found: template data collection forms; data extracted from included studies; data used for all analyses; analytic code; any other materials used in the review.	No analytic code or separate data files were generated for this review. All data extracted from included studies and materials used for synthesis are presented within the main manuscript and its appendices. These include the summary tables, risk of bias assessments, and search strategy details. No additional materials are publicly archived.

From: Page MJ, McKenzie JE, Bossuyt PM, Boutron I, Hoffmann TC, Mulrow CD, et al. The PRISMA 2020 statement: an updated guideline for reporting systematic reviews. BMJ 2021;372:n71. doi: 10.1136/bmj.n71. This work is licensed under CC BY 4.0. To view a copy of this license, visit https://creativecommons.org/licenses/by/4.0/
